# Supplementary material for: Mapping Plasmodium transitions and interactions in the Anopheles female
Source: Nature. 2025 Oct 22;648(8093):451–8. doi: 10.1038/s41586-025-09653-0 (PMC12695668; doi:10.1038/s41586-025-09653-0)
Supplement: Supplementary file 1 — Supplementary Note, Supplementary Figures, legends for Supplementary Tables 1–8, legends for Supplementary Videos 1–3 and Supplementary References. [file 41586_2025_9653_MOESM1_ESM.pdf]

nature portfolio

https://doi.org/10.1038/s41586-025-09653-0

Supplementary information

Mapping *Plasmodium* transitions and interactions in the *Anopheles* female

In the format provided by the authors and unedited

SI Guide

Mapping *Plasmodium* transitions and interactions in the *Anopheles* female

Yan Yan[1*], Lisa H. Verzier[1*], Elaine Cheung[1*], Federico Appetecchia[1], Sandra March[2,3,4,5], Ailsa R. Craven[1], Esrah Du[1], Alexandra S. Probst[1], Tasneem A. Rinvee[1], Laura E. de Vries[1], Jamie Kauffman[1], Sangeeta N. Bhatia[2,3,4,5,6], Elisabeth Nelson[1], Naresh Singh[1], Duo Peng[1,7], W. Robert Shaw[1,8], Flaminia Catteruccia[1,8,#]

[1] Department of Immunology and Infectious Disease, Harvard T. H. Chan School of Public Health, Boston, MA 02115, USA.
[2] Howard Hughes Medical Institute, Cambridge, MA 02139, USA.
[3] Institute for Medical Engineering and Science, Massachusetts Institute of Technology (MIT), Cambridge, MA 02139, USA.
[4] David H. Koch Institute for Integrative Cancer Research, MIT, Cambridge, MA 02139, USA.
[5] Broad Institute of MIT and Harvard, Cambridge, MA 02139, USA.
[6] Wyss Institute at Harvard University, 201 Brookline Ave, Boston, MA 02215, USA
[7] The Chan Zuckerberg Biohub, San Francisco, CA 94158, USA
[8] Howard Hughes Medical Institute, Boston, MA 02115, USA.

* These authors contributed equally
[#] Corresponding author: fcatter@hsph.harvard.edu

Table of Contents

Supplementary Note	3
Supplementary Figures	8
Supplementary Tables	9
Supplementary Videos	9
Supplementary References	10

Supplementary Note

Parasite sequencing depth requirements

Each replicate of the eight samples was mixed equally and initially sequenced on an Illumina iSeq 100 to determine the percentage of reads mapping to parasites relative to mosquito cells. As stated in the methods, around 0.05%, 0.1%, 0.6%, and 1.3% reads mapped to parasites at 36h, 2d, 4d and 7d pi, respectively. Based on hemocytometer counts, we estimated that each sample loaded onto the 10X chip contained an average of 380 parasites, resulting in an estimated total of 3,040 parasites per time point (from two metabolic conditions and four replicates). Based on these estimates, we selected two NovaSeq S4 flow cells, which were expected to yield a minimum of 12 billion reads (Broad Institute). This would provide an estimated 6-156 million reads mapping to parasites at 36h to 7d pi, corresponding to an estimated reading depth of 1,974–51,316 reads per parasite at 36h to 7d pi.

Canonical midgut cell types and functional validation in hematophagous mosquitoes

The mosquito midgut is divided into anterior and posterior regions, with the latter making up the bulk of the organ and serving as the site for blood meal storage and digestion[1]. Situated at the anterior-most end of the midgut, the proventriculus (PV), also known as the cardia, is a distinct structure involved in immune responses in adult mosquitoes[2,3]. Data on midgut cell types in hematophagous mosquitoes is relatively sparse (reviewed by Hixson *et al.*[4]) but is extensively available from studies of *Drosophila melanogaster*[5-8], and has been used to annotate cell types of other mosquito midgut scRNA publications. Canonically, these include intestinal stem cells (ISCs), undifferentiated enteroblasts (EBs), and the terminally differentiated enterocytes (ECs), enteroendocrine cells (EEs), and visceral muscle cells (VMs).

ISCs are the only replicative cells in the midgut epithelium, underpinning both tissue homeostasis and regenerative capacity. Located basally, with no direct access to the gut lumen, ISCs undergo either symmetrical—yielding two self-renewing ISCs—or asymmetrical division, producing one ISC and one differentiating daughter cell[9-11]. The existence of such stem cells in mosquito midguts was long debated but active mitosis has since been imaged in the midguts of *Anopheles* mosquitoes[12-14]. A recent study extensively characterized external stimuli, such as a blood meal or bacterial infection, which trigger ISC division in *An. gambiae* and *Ae. aegypti*[14].

Following asymmetric division, the daughter cell fated for differentiation may adopt one of two trajectories: it can become an EB—an intermediate, undifferentiated cell that will subsequently differentiate into an EC[11,12]—or a pre-EE that will mature into a functional EE[15]. Although EEs have been described in midgut images for decades[16-18], recognized by their abundant vesicles and basolateral exocytosis, their functional roles remain poorly characterized. A subset of these cells expresses neuropeptide F, a hormone implicated in stimulating feeding behaviour in *Ae. aegypti*[19], suggesting that EEs may serve as key regulators of host attraction and gut-brain communication.

ECs constitute the majority of the epithelium in both the anterior and posterior midgut. These large, columnar cells exhibit microvilli on their luminal surface, supporting their roles in digestive enzyme secretion and nutrient absorption[12,16,20]. ECs are polyploid, a feature particularly accentuated in *An. gambiae*, where nuclear content can reach up to 256n following a blood meal[14]. Their function varies according to their location, with anterior ECs being responsible for sugar digestion and absorption while the posterior ECs mainly produce enzymes for blood meal digestion and uptake amino acids[1,3,20].

Although not epithelial in origin, VMs are essential for midgut integrity. A lattice of longitudinal and circular muscle fibers envelops the basal surface of the midgut epithelium[21], enabling coordinated peristalsis and contributing to the mechanical plasticity of the organ during blood meal intake and digestion.

Mosquito scRNA cell cluster annotation

Mosquito single-cell data were annotated using a combination of single-cell (sc) and single-nucleus (sn) midgut studies, alongside bulk RNA-seq data from *Anopheles* and functional studies primarily conducted in *D. melanogaster* (reviewed by Zhang and Edgar[8]). First, cell localization was scored based on bulk RNA-seq expression profiles of distinct regions of the digestive tract, which includes the proventriculus (PV), and the anterior and posterior midguts[1]. This analysis led to the identification of cluster 10 as PV (Extended Data Fig. 6c).

We then compared our cluster markers with those reported in sn or scRNA-seq studies of *D. melanogaster*, *Ae. aegypti* and *An. gambiae*[5,6,22,23]. Given the comprehensiveness of the Fly Cell Atlas, which encompasses all *D. melanogaster* cell types, we limited the reference dataset to midgut-relevant populations: ISCs, EBs, VMs, EEs, ECs (anterior and posterior), cardia or PV, along with likely contaminants such as hemocytes (HC), fat body (FB), Malpighian tubule cells, and tracheal cells. By overlapping the expression of reference marker genes with our cluster marker genes (Supplementary Table 5), we observed a robust signal for EE in mosquito cell clusters 8, 9, 12, and 13; ISC/EB in

cluster 4; and VM in cluster 14. Signal from HC and FB remained mainly confined to the smallest cluster 15, consistent with their status as contaminants. Markers for Malpighian tubules, cardia, PV, and tracheal cells were largely absent, including in the previously annotated PV cluster. The remaining clusters were presumed ECs as they express EC-associated markers, even though expression of these markers was broadly distributed across multiple clusters.

Functional enrichment of each cluster was next used to validate cluster annotation (Supplementary Table 5). All EE-annotated clusters showed enrichment in biological processes related to cell communication and cell–cell signaling, consistent with their endocrine function. The ISC/EB cluster was enriched for the terms 'mitotic cell cycle' and 'DNA replication', reflecting its proliferative capacity, while the VM cluster showed enrichment for genes involved in sarcomere organization and muscle contraction. The PV cluster expressed high levels of genes associated with humoral immune response, in agreement with previous studies and its anticipated role in anti-microbial peptide production[1-3,6]. As expected for a cluster comprising two distinct cell types, the functional enrichment profile of the HC/FB cluster was less informative. Clusters 0, 1, 3, and 7, which showed high posterior scores, were enriched in proteolysis and metalloaminopeptidase activity, consistent with pEC identity. Clusters 2 and 5, both with high anterior scores, were enriched for heme-binding proteins—hallmarks of aECs[1]—and were annotated accordingly. Interestingly, cluster 6 showed a high posterior score but was enriched in sugar transporters, contrary to the notion that such function is restricted to the anterior midgut. This may reflect a greater investment of *An. gambiae* on carbohydrate digestion and absorption in the posterior midgut compared to *Ae. aegypti*, and the cluster was annotated as pEC[1]. Finally, cluster 11 was enriched for genes involved in amide and sulfur compound metabolism, but these functions have not been clearly linked to a specific midgut cell type.

Finally, we validated cluster annotations with established markers reported in the literature (Extended Data Fig. 6b). Delta and klumpfuss (klu)—canonical markers of ISCs and EBs, respectively[11,24]—were only weakly detected in our dataset, though headcase[25], a known ISC marker, emerged as one of the top five markers in the ISC cluster, supporting its annotation. The VM cluster showed high expression of myosin and actin genes, while the HC/FB cluster expressed SPARC and lipophorin, consistent with their known marker profiles[22]. All pEC clusters showed high levels of nubbin[5,8,23,26] and carboxypeptidase A, with low levels of gambicin. In contrast, aEC and PV clusters exhibited strong expression of gambicin; aECs also expressed sugar transporters and digestive enzymes, while PV was marked by a distinct eupolytin. EE clusters expressed prospero, a well-established EE marker in *D. melanogaster* and *Ae. aegypti*[5,23,26], as well as bruno 2, identified as an

EE marker in the latter species. Cluster 11 expressed prospero but lacked expression of most other EE markers, including bruno 2, and was therefore annotated as EE-like.

Together, this single-cell transcriptomic analysis enabled systematic identification and characterization of midgut cell types. For the first time in mosquito midgut studies, spatial positioning of individual cells and clusters was inferred from empirical data[1], revealing an anterior EE cluster previously identified through imaging[17,18].

Supplementary Figures

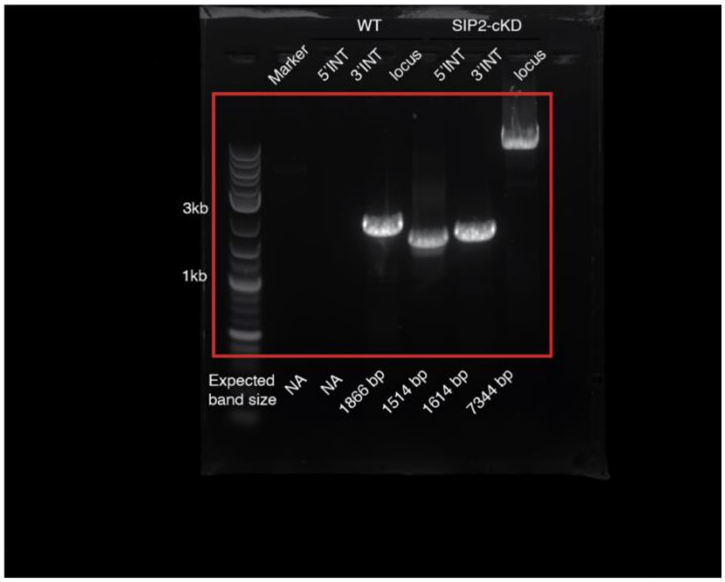

Supplementary Figure 1. Uncropped gel image from Extended Data Figure 5b, showing PCR amplifications of the 5' and 3' integrated regions (5'INT and 3'INT) and locus in WT NF54 and PfSIP2-cKD parasites. The red rectangles show the cropping location.

Supplementary Tables

Supplementary Table 1: Percentage of reads mapped to the genome of *An. gambiae* and *P. falciparum*, and quality control criteria for each sample.

Supplementary Table 2: Pseudobulk gene expression and functional enrichment of *P. falciparum*.

Supplementary Table 3: Marker genes for each cluster of the integrated *P. falciparum* midgut stages dataset.

Supplementary Table 4: Gene model of *P. falciparum* midgut stages from this study.

Supplementary Table 5: Top 100 marker genes and corresponding functional enrichment of each cluster of the integrated *An. gambiae* midgut cells and proportion across timepoints.

Supplementary Table 6: Pseudobulk differential expression analysis comparing mosquito cells in ds*EcR* and ds*GFP* treatment at each time point.

Supplementary Table 7: Pseudobulk gene expression and functional enrichment of *An. gambiae* midgut cells across time points post blood meal.

Supplementary Table 8: Primer information.

Supplementary Videos

Supplementary Video 1: 3D rendering of the Extended Data Figure 7f illustrating an ookinete that has left a Pfs25 trail into a large cell reminiscent of an enterocyte. DNA was labelled with DAPI (cyan), VM with phalloidin (grey), ISC with centrin (magenta) and parasite with Pfs25 (green).

Supplementary Video 2: Basal to lumen video of successive slices from the Fig 5a Z-stack illustrating the muscle stretch around and oocyst. DNA was labelled with DAPI (cyan), VM with phalloidin (grey), and ISC with centrin (magenta). Scale bar: 10 μm.

Supplementary Video 3: Lumen to basal video of successive slices from the Fig 5c Z-stack illustrating the muscle lattice encasing an oocyst. DNA was labelled with DAPI (cyan), and VM with phalloidin (grey).

Supplementary References

1 Hixson, B. *et al.* A transcriptomic atlas of *Aedes aegypti* reveals detailed functional organization of major body parts and gut regional specializations in sugar-fed and blood-fed adult females. *Elife* **11** (2022). https://doi.org:10.7554/eLife.76132

2 Vizioli, J. *et al.* Gambicin: a novel immune responsive antimicrobial peptide from the malaria vector *Anopheles gambiae*. *Proc Natl Acad Sci U S A* **98**, 12630-12635 (2001). https://doi.org:10.1073/pnas.221466798

3 Warr, E., Aguilar, R., Dong, Y., Mahairaki, V. & Dimopoulos, G. Spatial and sex-specific dissection of the *Anopheles gambiae* midgut transcriptome. *BMC Genomics* **8**, 37 (2007). https://doi.org:10.1186/1471-2164-8-37

4 Hixson, B., Taracena, M. L. & Buchon, N. Midgut epithelial dynamics are central to mosquitoes' physiology and fitness, and to the transmission of vector-borne disease. *Front Cell Infect Microbiol* **11**, 653156 (2021). https://doi.org:10.3389/fcimb.2021.653156

5 Hung, R. J. *et al.* A cell atlas of the adult *Drosophila* midgut. *Proc Natl Acad Sci U S A* **117**, 1514-1523 (2020). https://doi.org:10.1073/pnas.1916820117

6 Li, H. *et al.* Fly Cell Atlas: A single-nucleus transcriptomic atlas of the adult fruit fly. *Science* **375**, eabk2432 (2022). https://doi.org:10.1126/science.abk2432

7 Hung, R. J., Li, J. S. S., Liu, Y. & Perrimon, N. Defining cell types and lineage in the *Drosophila* midgut using single cell transcriptomics. *Curr Opin Insect Sci* **47**, 12-17 (2021). https://doi.org:10.1016/j.cois.2021.02.008

8 Zhang, P. & Edgar, B. A. Insect Gut Regeneration. *Cold Spring Harb Perspect Biol* **14** (2022). https://doi.org:10.1101/cshperspect.a040915

9 Ohlstein, B. & Spradling, A. The adult *Drosophila* posterior midgut is maintained by pluripotent stem cells. *Nature* **439**, 470-474 (2006). https://doi.org:10.1038/nature04333

10 O'Brien, L. E., Soliman, S. S., Li, X. & Bilder, D. Altered modes of stem cell division drive adaptive intestinal growth. *Cell* **147**, 603-614 (2011). https://doi.org:10.1016/j.cell.2011.08.048

11 Joly, A. & Rousset, R. Tissue adaptation to environmental cues by symmetric and asymmetric division modes of intestinal stem cells. *Int J Mol Sci* **21** (2020). https://doi.org:10.3390/ijms21176362

12 Baton, L. A. & Ranford-Cartwright, L. C. Morphological evidence for proliferative regeneration of the *Anopheles stephensi* midgut epithelium following *Plasmodium falciparum* ookinete invasion. *J Invertebr Pathol* **96**, 244-254 (2007). https://doi.org:10.1016/j.jip.2007.05.005

13 Janeh, M., Osman, D. & Kambris, Z. Damage-induced cell regeneration in the midgut of *Aedes albopictus* mosquitoes. *Sci Rep* **7**, 44594 (2017). https://doi.org:10.1038/srep44594

14 Taracena-Agarwal, M. L. *et al.* The midgut epithelium of mosquitoes adjusts cell proliferation and endoreplication to respond to physiological challenges. *BMC Biol* **22**, 22 (2024). https://doi.org:10.1186/s12915-023-01769-x

15 Zeng, X. & Hou, S. X. Enteroendocrine cells are generated from stem cells through a distinct progenitor in the adult *Drosophila* posterior midgut. *Development* **142**, 644-653 (2015). https://doi.org:10.1242/dev.113357
16 Baton, L. A. & Ranford-Cartwright, L. C. *Plasmodium falciparum* ookinete invasion of the midgut epithelium of *Anopheles stephensi* is consistent with the Time Bomb model. *Parasitology* **129**, 663-676 (2004). https://doi.org:10.1017/s0031182004005979
17 Baia-da-Silva, D. C. *et al.* Microanatomy of the American malaria vector *Anopheles aquasalis* (Diptera: Culicidae: Anophelinae) midgut: ultrastructural and histochemical observations. *J Med Entomol* **56**, 1636-1649 (2019). https://doi.org:10.1093/jme/tjz114
18 Brown, M. R., Raikhel, A. S. & Lea, A. O. Ultrastructure of midgut endocrine cells in the adult mosquito, *Aedes aegypti*. *Tissue Cell* **17**, 709-721 (1985). https://doi.org:10.1016/0040-8166(85)90006-0
19 Dou, X., Chen, K., Brown, M. R. & Strand, M. R. Reciprocal interactions between neuropeptide F and RYamide regulate host attraction in the mosquito *Aedes aegypti*. *Proc Natl Acad Sci U S A* **121**, e2408072121 (2024). https://doi.org:10.1073/pnas.2408072121
20 Hecker, H. Structure and function of midgut epithelial cells in *Culicidae* mosquitoes (Insecta, Diptera). *Cell Tissue Res* **184**, 321-341 (1977). https://doi.org:10.1007/BF00219894
21 Park, S. S. & Shahabuddin, M. Structural organization of posterior midgut muscles in mosquitoes, *Aedes aegypti* and *Anopheles gambiae*. *J Struct Biol* **129**, 30-37 (2000). https://doi.org:10.1006/jsbi.1999.4208
22 Raddi, G. *et al.* Mosquito cellular immunity at single-cell resolution. *Science* **369**, 1128-1132 (2020). https://doi.org:10.1126/science.abc0322
23 Cui, Y. & Franz, A. W. E. Heterogeneity of midgut cells and their differential responses to blood meal ingestion by the mosquito, *Aedes aegypti*. *Insect Biochem Mol Biol* **127**, 103496 (2020). https://doi.org:10.1016/j.ibmb.2020.103496
24 Korzelius, J. *et al.* The WT1-like transcription factor Klumpfuss maintains lineage commitment of enterocyte progenitors in the *Drosophila* intestine. *Nat Commun* **10**, 4123 (2019). https://doi.org:10.1038/s41467-019-12003-0
25 Resende, L. P., Truong, M. E., Gomez, A. & Jones, D. L. Intestinal stem cell ablation reveals differential requirements for survival in response to chemical challenge. *Dev Biol* **424**, 10-17 (2017). https://doi.org:10.1016/j.ydbio.2017.01.004
26 Wang, S. *et al.* A cell atlas of the adult female *Aedes aegypti* midgut revealed by single-cell RNA sequencing. *Sci Data* **11**, 587 (2024). https://doi.org:10.1038/s41597-024-03432-8
