# Supplementary material for: Mapping Plasmodium transitions and interactions in the Anopheles female
Source: Nature. 2025 Oct 22;648(8093):451–8. doi: 10.1038/s41586-025-09653-0 (PMC12695668; doi:10.1038/s41586-025-09653-0)
Supplement: Supplementary file 2 — Reporting Summary [file 41586_2025_9653_MOESM2_ESM.pdf]

nature portfolio

Corresponding author(s): Lisa Verzier, Flaminia Catteruccia

Last updated by author(s): Aug 15, 2025

Reporting Summary

Nature Portfolio wishes to improve the reproducibility of the work that we publish. This form provides structure for consistency and transparency in reporting. For further information on Nature Portfolio policies, see our Editorial Policies and the Editorial Policy Checklist.

Statistics

For all statistical analyses, confirm that the following items are present in the figure legend, table legend, main text, or Methods section.

n/a	Confirmed	
☐	☒	The exact sample size (*n*) for each experimental group/condition, given as a discrete number and unit of measurement
☐	☒	A statement on whether measurements were taken from distinct samples or whether the same sample was measured repeatedly
☐	☒	The statistical test(s) used AND whether they are one- or two-sided<br>*Only common tests should be described solely by name; describe more complex techniques in the Methods section.*
☐	☒	A description of all covariates tested
☐	☒	A description of any assumptions or corrections, such as tests of normality and adjustment for multiple comparisons
☐	☒	A full description of the statistical parameters including central tendency (e.g. means) or other basic estimates (e.g. regression coefficient) AND variation (e.g. standard deviation) or associated estimates of uncertainty (e.g. confidence intervals)
☐	☒	For null hypothesis testing, the test statistic (e.g. *F*, *t*, *r*) with confidence intervals, effect sizes, degrees of freedom and *P* value noted<br>*Give P values as exact values whenever suitable.*
☒	☐	For Bayesian analysis, information on the choice of priors and Markov chain Monte Carlo settings
☒	☐	For hierarchical and complex designs, identification of the appropriate level for tests and full reporting of outcomes
☒	☐	Estimates of effect sizes (e.g. Cohen's *d*, Pearson's *r*), indicating how they were calculated

Our web collection on statistics for biologists contains articles on many of the points above.

Software and code

Policy information about availability of computer code

Data collection	Our single cell library preparation was tested on the Illumina iSeq100 before running on two NovaSeq S4 flow cells for deep sequencing.
Data analysis	Single-cell analysis pipeline is described in the Code Repository. All other analysis can be found in the Methods section of the manuscript. The following software are used in this study: 10X Cell Ranger software v7.0.1, Python v3.10, Scanpy v1.9.1, Scrublet v0.2.3, SeuratDisk (v0.9021), Seurat (v5.0.2), Pegasus (v1.8.1) bioconductor-deseq2 (v1.42.0), GoProfiler (https://biit.cs.ut.ee/gprofiler/gost), R (v4.3.2), RStudio (v2023.9.1.494), Monocle3 (v1.3.4), scDblFinder(v1.16.0), OocystMeter (https://www.biorxiv.org/content/10.1101/2025.06.28.662088v1), ZEN (v3.10), Fiji (v2.14.0), GraphPad Prism (v10.1.1).

For manuscripts utilizing custom algorithms or software that are central to the research but not yet described in published literature, software must be made available to editors and reviewers. We strongly encourage code deposition in a community repository (e.g. GitHub). See the Nature Portfolio guidelines for submitting code & software for further information.

Data

Policy information about availability of data

All manuscripts must include a data availability statement. This statement should provide the following information, where applicable:

- Accession codes, unique identifiers, or web links for publicly available datasets
- A description of any restrictions on data availability
- For clinical datasets or third party data, please ensure that the statement adheres to our policy

Reference genomes from P. falciparum and An. gambiae were obtained from PlasmoDB (https://plasmodb.org/plasmo/app) and VectorBase (https://vectorbase.org/vectorbase/app) respectively. The published datasets were obtained from the Malaria Cell Atlas website (www.malariacellatlas.org) and from the GEO repository, accession number GSE222586. The source data for the single cell analysis are accessible from the GEO repository, accession number GSE284537. Additional source data, including mosquito infection data, are deposited into Harvard Dataverse repository (https://doi.org/10.7910/DVN/DCNUFV).

Research involving human participants, their data, or biological material

Policy information about studies with human participants or human data. See also policy information about sex, gender (identity/presentation), and sexual orientation and race, ethnicity and racism.

Reporting on sex and gender	N/A: Donors were anonymized so sex and gender are not known.
Reporting on race, ethnicity, or other socially relevant groupings	N/A: Donors were anonymized so ethnicity is not known.
Population characteristics	N/A: Donors were anonymized so any population characteristics are not known.
Recruitment	N/A: No recruitment is involved in the study.
Ethics oversight	N/A

Note that full information on the approval of the study protocol must also be provided in the manuscript.

Field-specific reporting

Please select the one below that is the best fit for your research. If you are not sure, read the appropriate sections before making your selection.

☒ Life sciences ☐ Behavioural & social sciences ☐ Ecological, evolutionary & environmental sciences

For a reference copy of the document with all sections, see nature.com/documents/nr-reporting-summary-flat.pdf

Life sciences study design

All studies must disclose on these points even when the disclosure is negative.

Sample size	We performed four independent biological replicates for our single cell data collection to collect enough parasites, due to the difference in number of parasites compared to mosquito cells (Graumans et al 2020 Trends in parasitology, PMID: 32620501), and account for biological variation inherent to an infection model. Parasite growth assays and mosquito infection experiments were all performed in triplicate, unless specified, as is the norm in the field (Probst et al. 2025 Nature, PMID: 40399670).
Data exclusions	No data were excluded.
Replication	2-4 biological replicates were performed and all replicate attempts were successful.
Randomization	Mosquitoes were collected as pupae and randomly assigned to different cages representing distinct treatment groups. Mosquitoes were then randomly aspirated from cages for double-stranded RNA injection, dissection for single-cell isolation, oocyst and sporozoite quantification, or microscopy analysis. For the asexual-stage growth assay, PfSIP2-cKD parasites were randomly assigned to wells containing different drugs.
Blinding	Blinding was not necessary as all mosquito infections were analyzed using the software OocystMeter.

Reporting for specific materials, systems and methods

We require information from authors about some types of materials, experimental systems and methods used in many studies. Here, indicate whether each material, system or method listed is relevant to your study. If you are not sure if a list item applies to your research, read the appropriate section before selecting a response.

Materials & experimental systems

n/a	Involved in the study
	☒ Antibodies
	☒ Eukaryotic cell lines
☒	Palaeontology and archaeology
	☒ Animals and other organisms
☒	Clinical data
☒	Dual use research of concern
☒	Plants

Methods

n/a	Involved in the study
☒	ChIP-seq
☒	Flow cytometry
☒	MRI-based neuroimaging

Antibodies

Antibodies used

Centrin-1 antibody, Kerafast, EBC004; Armadillo antibody, DSHB, N27A1; pfs25 antibody, BEI, MRA28, Clone 4B7, Lot#70013640; Phalloidin eFluor 660, Ebioscience, 50-6559-05; PfHSP70, antibodies-online, ABIN361730; goat anti-rabbit IgG 546, Invitrogen, A-11035; goat anti-rabbit IgG AlexaFluor 488, Invitrogen, A-11008; goat anti-mouse IgG AlexaFluor 488, Invitrogen, A-11001; goat anti-mouse AlexaFluor 568, Invitrogen, A-11004. DAPI, Millipore Sigma, D9542-5MG.

Validation

Centrin-1 antibody validation via co-staining with Armadillo for specificity staining progenitor cells. See Dinglasan et al. PNAS. 2007 for pfs25 staining. Spradling and Ohlstein. Nature. 2005 for armadillo staining. Chazotte. Cold Spring Harb Protoc. 2010. for phalloidin. Goel et al. Nature medicine 2015 for HSP70.

Eukaryotic cell lines

Policy information about cell lines and Sex and Gender in Research

Cell line source(s)

NF54 P. falciparum cell line from BEI Resources (MRA-1000), and field isolates ART29 and P5 (Paton, D. G. et al. 2022 PLoS Pathog)

Authentication

Parasite line was authenticated using nested PCR protocol with primers specific to P. falciparum.

Mycoplasma contamination

All P. falciparum strains were confirmed to be free of mycoplasma contamination.

Commonly misidentified lines
(See ICLAC register)

Name any commonly misidentified cell lines used in the study and provide a rationale for their use.

Animals and other research organisms

Policy information about studies involving animals; ARRIVE guidelines recommended for reporting animal research, and Sex and Gender in Research

Laboratory animals

Anopheles gambiae (G3 strain), An. coluzzii dervied from the field (Adams, K. L. et al. 2023 PLoS Pathog), and An. stephensi SDA-500. One-day-old female mosquitoes were used for double-stranded RNA injection, and 4-7-day-old female mosquitoes were used for other experiments.

Wild animals

No wild animals were used in the study.

Reporting on sex

Only female mosquitoes used, as males do not feed on blood and therefore do not carry P. falciparum.

Field-collected samples

No field collected samples were used in the study.

Ethics oversight

Lower invertebrates, such as mosquitoes, are exempt from regulations governing the use of animals in research.

Note that full information on the approval of the study protocol must also be provided in the manuscript.

Plants

Seed stocks	N/A
Novel plant genotypes	N/A
Authentication	N/A
